# Chickpea Seed Flours Improve the Nutritional and the Antioxidant Profiles of Traditional Shortbread Biscuits: Effects of In Vitro Gastrointestinal Digestion

**DOI:** 10.3390/antiox13010118

**Published:** 2024-01-17

**Authors:** Cristina Delgado-Andrade, Raquel Olías, Mari Carmen Marín-Manzano, Isabel Seiquer, Alfonso Clemente

**Affiliations:** Departamento Nutrición y Producción Animal Sostenible, Estación Experimental del Zaidín, Consejo Superior de Investigaciones Científicas (CSIC), San Miguel 101, Armilla, 18100 Granada, Spain; raquel.olias@eez.csic.es (R.O.); maricarmen.marin@eez.csic.es (M.C.M.-M.); alfonso.clemente@eez.csic.es (A.C.)

**Keywords:** chickpea biscuits, in vitro gastrointestinal digestion, protein digestibility, amino acid bioaccessibility, polyphenols, antioxidant activity

## Abstract

Functional gluten-free biscuits enriched with commercial and landrace non-commercial chickpea flours were designed and compared with a traditional shortbread biscuit. They were analyzed in sensory attributes, amino acid profile, and antioxidant properties. Subsequently, the biscuits were digested in vitro to evaluate protein hydrolysis, amino acid bioaccessibility, phenolic compounds release, and antioxidant markers. The presence of chickpea flours provided golden color and heightened biscuit hardness and fracturability (especially in non-commercial), increasing crispness and reducing brittleness. The protein hydrolysis was similar among samples (≈15%), except for one of the non-commercial (≈20%). Amino acids such as arginine, phenylalanine, leucine, tyrosine, and lysine exhibited the highest bioaccessibilities. Incorporating chickpea flour improved the antioxidant activity and polyphenol content in undigested samples and bioaccesible fractions, with higher levels of p-coumaric and ferulic acids after digestion, regardless of the chickpea seed. Non-commercial flours increased the presence of resveratrol and/or catechin in the bioaccessible fraction. Antioxidant action assessed in the Caco-2 cell line showed that the protective effect against reactive oxygen species (ROS) generation did not always correlate with the in vitro antioxidant capacity. Our data support that the inclusion of chickpea flours in the formulation of functional biscuits provides the consumer with products of added nutritional value with attractive organoleptic features.

## 1. Introduction

Biscuits are a very popular and versatile snacks appreciated for their convenience, diverse flavors, widespread availability, extended shelf life, and affordability. The global market biscuit size was evaluated at USD 101.15 billion in 2022 and is projected to grow to 148.56 billion by 2023 [1]. The traditional recipe typically combines cereal flours, sugars, and fats, which are blended and baked at high temperatures for short durations (up to 200 °C for less than 20 min) in order to minimize water content to below 10% and create an appealing golden-brown exterior. The biscuit market is continuously evolving in response to consumer demands, compelling producers to develop new formulations that either entirely or partially replace traditional cereal flours with alternatives deemed healthier or suitable for specific population groups. These new formulations include gluten-free options and incorporate innovative ingredients like spelt, kamut, teff, legumes, and various seeds intended to diverse lifestyles and dietary preferences [2]. Gluten-free products frequently suffer from poor technological quality, characterized by a low volume, lack of color, and tendency to crumble. Despite their significant variability in nutrient composition, many of these products lack proteins and instead contain high levels of fats [3]. Legume flour, with its high protein content and quality, proves to be an ideal ingredient for enhancing the nutritional value of bread and bakery products [4]. Ongoing efforts have been made to promote the consumption of legume grains by integrating them into various food products, including spaghetti [5], bread [6], cakes [7], or biscuits [8]. Specifically, chickpeas (*Cicer arietinum* L.) are notable for their high protein content and are valued for their excellent balance of essential amino acids, substantial levels of complex carbohydrates (resulting in a low glycemic index), antioxidant compounds such as polyphenols, vitamins, and minerals. Moreover, they contain relatively low levels of anti-nutritional factors [9]. Consequently, incorporating chickpea flour into bakery products could enhance the bioactive profile of new items. El-Gohery [9] found that substituting wheat and barley pretzels with 40% chickpea or 20% sweet lupine powder increased their total phenolic and total flavonoid contents while preserving or enhancing their sensory characteristics. However, it is crucial to note that processing can impact the polyphenol profile, consequently affecting the antioxidant properties and nutritional value of the reformulated food [10].

It is important to note that not all ingested nutrients and bioactive compounds are automatically bioavailable in the body. Throughout the digestive process, factors such as mastication, pH variations, and the activities of digestive enzymes in the mouth, stomach, and intestine can induce structural changes, chemical changes, and interactions between the different nutrients, influencing the bioaccessibility of these compounds [11]. Bioaccessibility can be described as the portion of a compound that is released from the food matrix in the gastrointestinal tract and is available for absorption in the intestine. Nutritionally, measuring bioaccessibility offers valuable insights essential for selecting food matrices that optimize the nutritional quality of final food products [12]. Therefore, in vitro gastrointestinal digestion (IVGD) methods serve as valuable tools for assessing the potential in vivo impact of any modifications made to a food product’s composition. These methods provide information on the protein digestibility and the bioaccessibility of various food components and their bioactivities.

The purpose of this investigation was to design functional gluten-free biscuits enriched with selected commercial and landrace non-commercial chickpea flours and to compare them with a traditional shortbread biscuit. The formulated biscuits were analyzed in sensory attributes, such as color and texture, amino acid profile, and antioxidant properties. The INFOGEST IVGD method was applied to evaluate protein hydrolysis and amino acid bioaccessibility, phenolic compounds release, and antioxidant actions of the formulated biscuits.

## 2. Materials and Methods

### 2.1. Reagents and Chemicals

Methanol was provided by VWR (Barcelona, Spain), while sodium bicarbonate, acetate sodium, galic acid, and hydrochloric acid (HCl) were acquired from Merck (Darmstad, Germany). The ABTS (2,2′-Azino-bis (3-ethylbenzothiazoline-6-sulfonic acid) diammonium salt was obtained from Amresco (Solon, OH, USA). 2,4,6-Tri(2-pyridyl)-s-triazine (TPTZ), and iron (III) chloride for the ferric-reducing power (FRAP) assay was from Fluka Chemicals (Fluka Chemicals, Madrid, Spain). Trolox ((±)-6-Hydroxy-2,5,7,8-tetramethylchromane-2-carboxylic acid), Folin–Ciocalteu reagent, all phenolic compounds, diethyl ether, methanol, α-amylase from human saliva (A1031), pepsin from porcine gastric mucosa (P6887), pancreatin from porcine pancreas (P7545), bile salts (B8756), sodium dodecyl sulfate (SDS), o-phthaldialdehyde (OPA), DL-dithiothreitol (DTT), L-serine, 4-(2-hydroxyethyl)-1-piperazineethanesulfonic acid) (HEPES), tert-butylhydroperoxide (t-BOOH), the cell culture media, and cell culture-grade chemicals were from Sigma-Aldrich (Sigma-Aldrich, St. Louis, MO, USA). The chemicals were all of high purity or analytical reagent grade. Bi-distilled deionized water (Milli-Q purification system, Millipore, Bedford, MA, USA) was used. Ingredients for biscuits’ preparation were purchased from local supermarkets.

### 2.2. Samples

An initial screening of chickpea ecotypes for the biscuit formulation was performed among 9 different seeds (3 commercials and 6 landrace non-commercials). According to the nutritional, polyphenol, and antioxidant profiles, 4 of the seeds (2 commercials and 2 non-commercials) were selected for the design of functional biscuits. The two commercial chickpea seeds (Kabuli type) were extra-quality and widely distributed in Spain, coming from Spanish protected geographical indications: *Fuentesauco* variety (named as Commercial 1, C1) and *Escacena* variety (named as Commercial 2, C2). The two landrace non-commercial chickpeas (Desi type) were provided by the National Center of Phylogenetic Resources (CRF) belonging to the National Institute for Agricultural and Food Research and Technology (INIA-CSIC), owing to a normalized agreement for material transference (num. SMTA-00AB19-00EB10-212229). They were grown in the facilities of the CRF at Alcalá de Henares (Madrid, Spain), and the selection of these cultivars was based on seed coat color and their cold resistance. The seeds were identified with their original codes assigned by the provider center (genbank number), but a simplified name was also given as follows: BGE041468 (named as Non-Commercial 1, NC1) and BGE050036 (named as Non-Commercial 2, NC2).

The four chickpea seeds were finely ground and placed in a polyethylene container, sealed under vacuum, and stored at 4 °C until used.

### 2.3. Preparation of Biscuits

A control biscuit (free of chickpea flour) was formulated according to the recipe described in AACC (American Association of Cereal Chemists) method 10–54 [13], with minor modifications. Ingredients were as follows: corn starch (51 g), powdered sucrose (13 g), unsalted butter (23 g), sodium bicarbonate (0.36 g), ammonium bicarbonate (0.18 g), salt (0.40 g), and distilled water (12.06 g). Various internal tests were conducted to establish the permissible quantity of chickpea flour within this formulation, based on both sensory and technological considerations. Finally, the functional biscuit included 30% of raw chickpea flour, with was included in the recipe replacing 30 g of corn starch with the different chickpea flours. The ingredients were thoroughly mixed, and the dough was rolled out to disks with a diameter of 6 cm and a thickness of 3 mm, and baked at 190 °C for 25 min in a conventional oven (UNE 400, Memmert GmbH, Schwabach, Germany). Biscuits were named Control, C1, C2, NC1, and NC2. Seven biscuits per batch and two batches per formulation were prepared. Three biscuits per batch were ground and mixed, and analytical determinations were performed at least in duplicate. For the color and texture tests, three biscuits per batch for each formulation were used. 

### 2.4. Color Measurements

The color of the biscuits was measured in accordance with CIE L*, a*, b*color measuring system with a HunterLab D25-9 optical sensor (Hunter Associates Laboratory, Reston, VA, USA). The illuminant and viewing geometry were D65 and 10°, respectively. Solid colors were named as follows: *L*^∗^ (black–white component, lightness), and the chromaticness coordinates, *a*^∗^ (+red to −green component) and *b*^∗^ (+yellow to −blue component). These values were used to calculate the chroma or saturation (C*), as C* = (*a*^⁎2^ + *b*^⁎2^)^1/2^, and the hue angle (h*), as h* = tan^−1^ (b^*^/a^*^). Hue angle values represent the degree of redness, yellowness, greenness, and blueness all together. The equipment was calibrated with a white ceramic tile (L*/93.80; a*/0.3156; b*/0.3319). Each color value reported was the mean of three determinations at 22–24 °C.

### 2.5. Texture Analysis

A three-point bending test was performed on three biscuits per batch using a texture analyzer (EZ-LX HS, Shimadzu, Columbia, MD, USA) equipped with a 50 kg load cell. Biscuit samples were placed on base beams with a distance of 4 cm between the two beams. A three-point bending rig was used with an HDP/3PB, knife-edge probe. The analyzer was set at test speed of 1 mm/s, posttest speed of 10 mm/s, trigger force of 20 g, and distance of 10 mm. The breaking force (N) of biscuits, also named as hardness, was recorded using the force-in-compression. In addition, the fracture stress (N/mm^2^) and the fracturability (mm), which were represented as the maximum force required to break the biscuit per unit area and the corresponding displacement at the maximum force, respectively, were chosen as parameters to evaluate texture.

### 2.6. Protein and Amino Acid Contents of Biscuits

The protein was analyzed using a general combustion method [14] (procedure 992:23) by using a LECO FP-2000 protein/nitrogen analyzer (Leco Instruments, Madrid, Spain) calibrated with EDTA (Dumas method). The nitrogen-to-protein conversion factor considered was 6.25%. Results are expressed as grams of protein/100 g of sample. Protein analysis showed values <0.3% in the Control biscuit. Then, due to the negligible protein content, this sample was excluded from later determinations related to amino acid analyses and degree of protein hydrolysis after digestion.

A Biochrom 30 amino acid analyzer, employing ion-exchange liquid chromatography and post-column continuous reaction with ninhydrin, was used for quantitative amino acid (aa) analysis. The ninhydrin derivatives eluted from the columns were monitored at 570 nm and 440 nm (specifically for proline). The resulting chromatograms provided information on the identity and quantity of aa present in the samples. The analysis of total aa content involved the hydrolysis of 50 mg with 5 mL of 6 N HCl containing 1% phenol. The resulting solutions were sealed in tubes under nitrogen and incubated at 110 °C for 24 h. Sulfur-containing aa, specifically cysteine and methionine, were quantified as methionine sulphone and cysteic acid following performic acid oxidation. The determination of tryptophan was not feasible due to its degradation during acid hydrolysis.

### 2.7. Antioxidant Profile and Total Phenolic Content of Biscuits

Before assessing antioxidant activity and total phenolic compounds (TPC), a chemical extraction was conducted following the method described by Pérez-Jiménez and Saura-Calixto [15]. Briefly, 0.150 g of sample was placed in a tube, and 6 mL of acidic methanol/water (50:50 *v*/*v*, pH 2) was added. The tube was thoroughly shaken at room temperature for 20 min and centrifuged at 2500× *g* for 10 min at 4 °C. Subsequently, the supernatant was recovered. To the residue, 4 mL of the same acidic methanol/water was added, and the shaking and centrifugation steps were repeated. The second methanolic extract was combined with the first one. For antioxidant activity and total phenolic content measurements, appropriate dilutions with distilled water were made when necessary.

*ABTS assay*. This method evaluates the capacity to scavenge free radicals and was carried out following the protocol outlined by Rufián-Henares and Delgado-Andrade [16] with slight modifications. The ABTS+· was produced by reacting 7 mM ABTS+· stock solution with 2.45 mM potassium persulphate and allowing the mixture to stand in the dark at room temperature for 12–16 h before use. The ABTS+· working solution (stable for 2 days) was diluted with a mixture of ethanol:water (50:50) to an absorbance of 0.70 ± 0.02 at 730 nm. For the analyses, 40 µL of the sample extract, blank or Trolox standard, and 200 µL of 5 mM pH 8.4 phosphate buffer were added with 60 µL of diluted ABTS+· solution. The absorbance reading was taken at 10 min using the microplate reader previously described. Aqueous solutions of Trolox were used for calibration (15–125 µM). Results were expressed as µmol equivalents of Trolox (TEAC)/g of sample. 

*FRAP assay.* The FRAP method is based on the reducing power of an antioxidant from ferric ion (Fe^3+^) to the ferrous ion (Fe^2+^). This electron transfer is directly proportional to the antioxidant activity. The method was performed according to Seiquer et al. [17]. The FRAP reagent was prepared daily by mixing 10 mM Fe^2+^-2,4,6-Tri(2-pyridyl)-1,3,5-triazine (TPTZ) with 40 mM HCl, 20 mM ferric chloride and 0.3 M acetate sodium buffer (pH 3.6) at the ratio of 1:1:10 *v*/*v*/*v*. For the analysis, 20 µL of the sample extract, blank, or Trolox standard was added to 280 µL of warmed FRAP reagent (37 °C) and incubated at 37 °C for 30 min in darkness, and the absorbance was read at 595 nm. The same aqueous solutions of Trolox previously mentioned were used for calibration. Results were expressed as µmol equivalents of Trolox (TEAC)/g of sample. 

*Total phenolic compounds (TPCs)*. The TPC was determined following the Folin–Ciocalteau colorimetric method [17]. For the analysis, 10 µL of the sample extract, blank, or gallic acid standard and 10 µL of Folin–Ciocalteau reagent were mixed and allowed to stand for 3 min. Then, 200 µL of sodium carbonate solution (75 g/L) was added; the volume was made up to 250 µL with Milli-Q water, mixed, and allowed to stand in the dark for 60 min. The absorbance was measured at 750 nm against a standard curve of gallic acid (25–250 mg/L) (y = 0.2508x − 0.0347; R^2^ = 0.999). Results were expressed as µmol equivalents of gallic acid (GAE)/g of sample.

### 2.8. IVGD of Biscuits

The IVGD assays followed the harmonized INFOGEST protocol [11,18] and were performed at least in triplicate for every biscuit tested. Enzyme activities and bile concentrations were determined following the procedures detailed earlier [11]. The digestion covered oral, gastric, and intestinal phases, reaching a final volume of 8 mL. In the oral phase, 1 g of crushed biscuit was combined with pre-warmed simulated saliva fluid and α-amylase (A1031; 96 U/mg). The mixture was then agitated at 80 rpm in a water bath at 37 °C for 2 min. In the gastric phase, the oral bolus was combined with simulated gastric fluid. The pH was adjusted to 3.0, and porcine pepsin (P6887; 3359 U/mg) was introduced to attain a final mixture containing 40,000 U. Subsequently, the mixture was promptly incubated in a water bath at 37 °C, with agitation at 80 rpm, for a duration of 2 h. After gastric digestion, the pH was adjusted to pH 7.0. In the intestinal phase, a combination of simulated intestinal fluid and 4000 U of pancreatin (P7545; 5.18 IU trypsin/mg) was introduced into the mixture, along with a 160 mM bile solution (B8756; 2.40 mmol of bile salts/g). The digesta underwent a 2 h incubation in a water bath at 37 °C with continuous mixing at 80 rpm. To halt the intestinal digestion, samples were promptly frozen in liquid nitrogen. Afterward, the samples were thawed on ice and subjected to centrifugation at 3220× *g* at 4 °C for 45 min. Supernatants (bioaccesible fractions after digestions, BFs) were used for the following analyses. The Control biscuit was used as a blank for the studies related to protein digestion since it was almost free of protein. 

### 2.9. Analyses Performed in the BFs of the In Vitro Digested Biscuits

#### 2.9.1. Degree of Protein Hydrolysis

The degree of protein hydrolysis (DH%) of the digested biscuit samples was evaluated spectrophotometrically at 340 nm. This measurement involved the reaction of primary amino groups using the OPA (o-phthaldialdehyde) [19]. The OPA reagent was prepared by dissolving 160 mg OPA in 4 mL ethanol. To this solution, a borate/SDS solution was added, consisting of 7.62 g di-sodium tetraborate decahydrate and 200 mg SDS in 150 mL deionized water. Then, 176 mg DTT was added, and the final volume of the solution was adjusted to 200 mL with distilled water. Free amino group concentrations were determined using a calibration curve based on L-serine (12.5–100 mg L^−1^), which exhibits a response close to the average response of amino acids in OPA reactions. DH% was estimated according to Equation (1) [20]:

DH % = [NH_2_ (final) − NH_2_ (initial)] × 100 / NH_2_ (acid) − NH_2_ (initial)] (1)

where NH_2_ (final) is the concentration of free amino groups in the digested sample after each phase, NH_2_ (initial) is the concentration of free amino groups before digestion, and NH_2_ (acid) is the total content of the completely hydrolyzed sample in 6 N HCl at 110 °C for 24 h. All measurements were carried out at least in triplicate for each digestion. 

#### 2.9.2. SDS-PAGE Analysis of Protein Profiles 

Using gradient 4–12% Bis-Tris pre-cast gels (Invitrogen, Barcelona, Spain) according to the manufacturer’s instructions, denaturing gel analyses of biscuit proteins before and after digestion were conducted. The running buffer employed was 2-N-morpholine-ethane sulphonic acid (NuPAGE MES, Invitrogen). As previously described by Olias et al. [21], before loading, the samples underwent reduction with DTT, and NuPAGE antioxidant was introduced into the upper buffer chamber to avert the re-oxidation of reduced proteins during electrophoresis (Expedeon, Harston, UK) or InstantBlue (Abcam, Cambridge, UK). Protein standards used in gel analyses included Mark12TM (Invitrogen, LC5677, Spain) with proteins in the range of 2.5 to 200 kDa or SeeBlue Plus2 pre-stained standards ranging from 3 to 198 kDa (Thermofisher, Waltham, MA, USA). 

#### 2.9.3. Amino Acid Released after IVGD

Free amino acid contents in the BFs after digestion were determined following deproteinization of the samples as previously described by Aristoy and Toldrá [22], with minor modifications. Briefly, 500 µL of TCA (20%) with 2 mM of norleucine as internal standard, per 500 µL of the soluble fraction, was added followed by centrifugation at 2700× *g* for 1 h at 5 °C. Supernatants were stored at −20 °C until analysis, using the same equipment and techniques previously described in the case of undigested biscuits. The free amino acid analysis was determined once for each digestion. 

#### 2.9.4. Phenolic Compounds Released after IVGD

Individual phenolic compounds in the BFs of digested biscuits were determined with the UPLC-ESI-MS/MS-QTOF method proposed by [23], with modifications. A liquid–liquid extraction was performed by adding 1 mL of diethyl ether to 0.5 mL of BF, and the resulting solution was frozen at −20 °C for 24 h, after which it was centrifuged for 15 min at 3220× *g*. The supernatant was then transferred to a separatory funnel, and three extractions were carried out using 1 mL of diethyl ether. Then, a spatula tip of anhydrous sodium sulfate was introduced to the combined organic extract, and the mixture underwent centrifugation for 15 min at 3220× *g*. The resulting clean supernatant was vacuum-dried at 30 °C. The dried extracts were collected with 0.5 mL methanol/water mixture (1:1), filtered through a 0.20 μm membrane filter, and passed to a chromatography vial for analysis. Chromatographic analysis of the polyphenols was performed at the Scientific Instrumentation Centre of the University of Granada using an Acquity UPLC H-Class with MS detection (Waters, Barcelona, Spain), and separations were achieved using a Waters Acquity UPLC HSS T3 column (100 mm × 2.1 mm, 1.8 μm particle size). Gradient elution was used for chromatographic separation, employing water +0.5% acetic acid as eluent A and acetonitrile + 0.5% acetic acid as eluent B. The flow rate was adjusted to 0.4 mL/min, the column temperature was kept at 45 °C, and the injection volume was set to 10 μL. The elution process began with 5% eluent B for the first 15 min, followed by a transition to 95% eluent B, concluding the total run time at 18 min. The mass spectrometry analysis was carried out using a Waters SYNAPT G2 HDMS Q-TOF high-resolution spectrometer. The instrument was operated using ESI ionization in the negative ion mode. The measurement range was 50–1200 atomic mass unit (amu). The identification and quantification of phenolic compounds was performed by comparing the negative masses recorded in previous research, using MassLynx V4 software (Waters Laboratory Informatics, Waters, 2010). Individual phenolic compounds were quantified by obtaining a series of solutions, with a concentration of 5–40 mol, of standard phenolic compounds with different retention times. For each phenolic compound selected, a calibration curve with R^2^ ≥ 0.997 was performed to ensure the linearity of the method. 

#### 2.9.5. Antioxidant Activity and TPC of Digested Biscuits

The antioxidant action was measured in the BFs using the ABTS and FRAP methods as previously described [16,17]. Similarly, TPC was determined according to the Folin–Ciocalteau colorimetric method mentioned above [17]. Results were expressed as µmol equivalents of Trolox (TEAC)/g of digested biscuit and as µmol equivalents of gallic acid (GAE)/g of digested biscuit, respectively.

#### 2.9.6. Reactive Oxygen Species (ROS) Generation in Caco-2 Cells

The antioxidant potential at the cellular level of the BFs obtained after IVGD was assessed. This evaluation involved measuring its impact on reactive oxygen species (ROS) generation in Caco-2 cells, following the methodology described by Borges et al. [24]. To guarantee cell viability, the fractions underwent purification via ultrafiltration, using a 30 kDa cut-off membrane (Amicon Ultra-15; Millipore, Darmstadt, Germany) to remove additional digestion enzymes and macromolecular compounds. Caco-2 cells were purchased through the Cell Bank of Granada University (Granada, Spain) from the European Collection of Cell Cultures (ECACC). The cells were cultured in 75 cm^2^ plastic flasks (Costar, Cambridge, MA, USA) through successive passages. The culture medium consisted of high-glucose Dulbecco’s modified minimal essential medium (DMEM), supplemented with heat-inactivated fetal bovine serum (FBS) at 10%, NaHCO_3_ at 3.7 g/L, nonessential amino acids at 1%, HEPES at 15 mM, bovine insulin at 0.1 UI/mL, and a 1% antibiotic–antimycotic solution. ROS determinations were conducted under both basal conditions and induced oxidative stress. Experiments were performed using BF:FSB-free DMEM (at a ratio of 1:2 *v*/*v*). Previous assays with the colorimetric MTT method (3-(4,5-dime thylthiazol-2-yl)-2.5-diphenyltetrazolium bromide, Roche, Mannheim, Germany) indicated that cell viability exceeded 90% under these conditions.

ROS generation was determined through the dichlorofluorescin (DCFH) assay. Cells were seeded in 96-well multiwell plates at a concentration of 10 × 10^4^ cells/mL (100 µL/well) and allowed to grow for 48 h. Following the removal of the spent medium, cells were preincubated with the BF for 2 h. Subsequently, cells were treated with DCFH at 20 µM and incubated for 1 h. The DCFH was then removed, and the FBS-free culture medium (for basal measurements) or t-BOOH at 20 mM (to induce oxidation) was added to the wells. Absorbance was measured at a wavelength of 485 nm excitation and 535 nm emission at 37 °C over a period of 0–70 min. In the presence of free radicals such as ROS, DCFH is oxidized into dichlorofluorescein (DCF) and emits fluorescence, which is measured to estimate ROS production. Results of ROS generation were expressed in fluorescence units.

### 2.10. Statistical Analyses

Statistical analyses were performed by using SPSS version 16.0 (SPSS Inc., Chicago, IL, USA) and Statgraphics Centurion XV (Herndon, VA, USA). The statistical significance of the variables was tested by applying analysis of variance (ANOVA one-way) and the mean comparisons was performed according to Tukey HSD test. When necessary, relationships between the different variables were evaluated by computing Pearson’s linear correlation coefficient. All statistical parameters were evaluated at *p* < 0.05 significance level.

## 3. Results and Discussion

### 3.1. Color and Texture Analyses in the Formulated Biscuits

**Figure 1:**
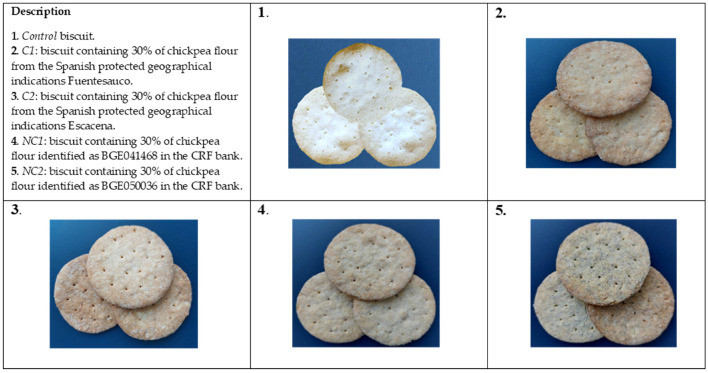
External appearance of formulated biscuits.

Figure 1 displays the external appearance of the formulated biscuits, with a detailed description of the specific chickpea ecotypes utilized in the recipe. The color of biscuits has a significant impact on their acceptance by consumers; hence, it is important to determine how recipe alteration affects biscuits appearance.

**Figure 2:**
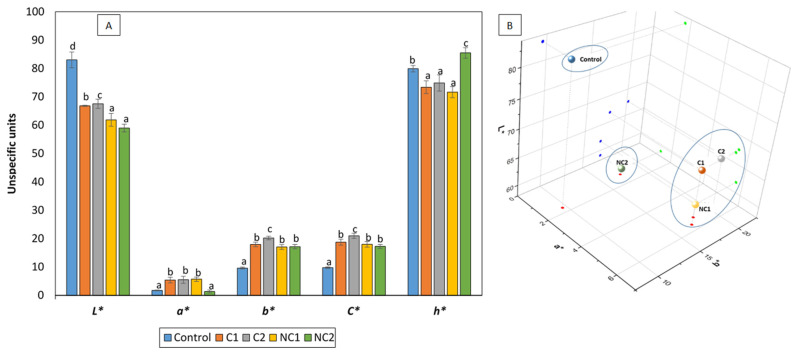
CIElab color of formulated biscuits (**A**) and 3D representation (**B**). h* is expressed in degrees. Data are expressed as mean values ± SD. Different letters within each parameter indicate significant differences between biscuits (*p* < 0.05).

The partial substitution of corn starch with different chickpea flours influenced the color formation of the biscuits, resulting in significantly lower L* values (reduced lightness, darker biscuits) and higher b* values (prevalence of golden tones) (Figure 2A). The higher mean protein content in the chickpea biscuits compared to traditional shortbread biscuits (8.66% vs. 0.23%, *p* < 0.05) contributed to an extended Maillard reaction during the baking process, thus increasing the development of browning products which may reduce L* and elevate the b* values. In fact, biscuit NC2, which had the highest protein content, was also the darkest. This phenomenon aligns with previous studies [25,26] that observed a similar effect after replacing cereal flour by chickpea flour in the formulation of biscuits. In addition, chickpea flour, due to its own yellow color, also promotes golden tones, increasing b* values [25]. Regarding the a* values, a shift towards reddish hues was noted in the presence of legumes, with the exception of NC2, which maintained a similarity to the control biscuit. The existing scientific literature consistently outlines the same pattern observed on the L* and b* values following the addition of chickpea flour to biscuit recipes. Conversely, the behavior of parameter a* seems to be more unpredictable and dependent on the presence of other ingredients, particularly polysaccharides [27]. As a consequence of the higher a* and b*, the C* values significantly increased in all biscuits enriched with chickpea flours, which is consistent with findings from other researchers [27]. The hue angle (h*) values indicate the extent of redness, yellowness, greenness, and blueness, with maximum values at 0, 90, 180, and 270°, respectively. Chickpea biscuits differed significantly from the control, and notable distinctions were observed among them. C1, C2, and NC1 displayed the lowest values, closer to reddish tones, while NC2 exhibited a value close to 90°, indicating a predominance of yellow and golden tones. The pattern reflected by the h* values aligns with the arrangement of the biscuits in the 3D color space (Figure 2B). Previous research has shown that biscuits with chickpea flour have a desirable golden-brown hue, which is attractive to consumers [27].

**Figure 3:**
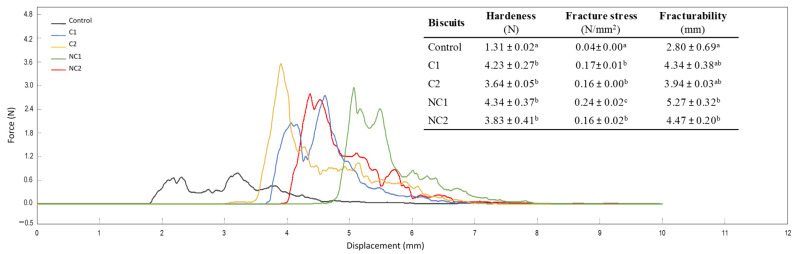
Three-point bending test curves and parameters selected to evaluate texture in formulated biscuits. Data are expressed as mean values ± SD. Different letters within each column indicate significant differences between biscuits (*p* < 0.05).

As reported in Figure 3, the addition of chickpea flours to the biscuit formulations resulted in variations in texture, a crucial quality aspect in bakery products that is linked to consumers’ perception of freshness [28]. The profile of the force–displacement curves clearly shows the distinct behavior of traditional shortbread biscuits compared to the new formulations with chickpea flours. The properties of hardness, fracture stress, and fracturability provide information on the firmness of the structure. Hardness is typically considered as an unfavorable attribute in biscuit products, whereas fracturability is associated with a pleasing sensory quality as long as it does not become excessive [26]. However, moderate hardness also indicates the firmness of the structure, which may be desirable for biscuits, preventing excessive fragility. 

The inclusion of chickpea flours resulted in heightened biscuit hardness, also indicated by the fracture stress values. Fracturability increased, particularly with the incorporation of non-commercial seeds (*p* < 0.05), providing greater crispness and less fracturable biscuits. These findings underscore a substantial enhancement in the texture of chickpea biscuits when compared with conventional shortbread ones, which are typically brittle and pose packaging challenges. Lu et al. [25] also demonstrated higher hardness and fracturability due to the inclusion of different percentages of chickpea flours in low-gluten wheat biscuits, whereas Schouten et al. [26] did not find substantial changes. This discrepancy highlights the need for further exploration and consideration of varying factors in the interaction between chickpea flour and biscuit properties. 

### 3.2. Effects of IVGD on Protein and Amino Acid Profiles 

Gluten-free biscuits often lack essential nutrients, particularly proteins. The incorporation of chickpea flour presents a promising approach to enhance their nutritional profile, as chickpeas typically feature higher protein contents than the cereals traditionally employed in biscuit production. Despite using equal amounts of meal in all biscuits (30%), variations in protein content were observed, with the non-commercial samples exhibiting notably higher values (Table 1).

**Figure 4:**
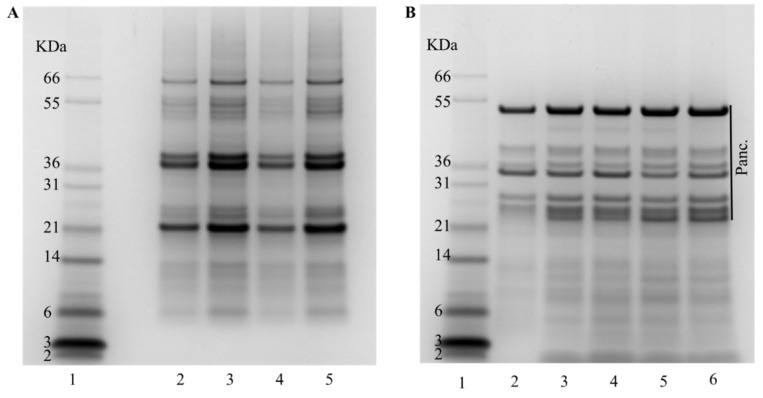
Protein profile of the biscuits before and after IVGD using an SDS-PAGE (4–12%) gel. (**A**) Before digestion. Lane 1: molecular markers; Lane 2: Control biscuit; Lane 3: C1; Lane 4: C2; Lane 5: NC1; Lane 6: NC2. (**B**) After digestion. Lane 1: molecular markers; Lane 2: Control biscuit; Lane 3: C1; Lane 4: C2; Lane 5: NC1; Lane 6: NC2.; Panc. refers to proteins present in Pancreatin.

To assess protein hydrolysis degree (DH) and amino acid availability, the IVGD of the different biscuits was performed using the conditions of the consensus model developed through the INFOGEST network [18]. The DH, measured by OPA, was consistently around 15% for most biscuits, with the exception of the biscuit made with NC1 flour that exhibited a higher DH of 20% (Table 1). Protein hydrolysis depends on the food matrix among other factors. In the case of biscuits, the combination of starch, protein, and fat forms a network structure that is fragmented along the digestion process, releasing the different nutrients. While variations in protein content among biscuits could potentially impact this network, as previously suggested by Lu et al. [25], it is improbable in our case, given the relatively minor differences observed and the consistent use of the same quantity of chickpea meal. The higher protein hydrolysis of the biscuits made with NC1 is probably due to the unique characteristics of these seeds. Despite an apparent similarity in the initial protein profile, as illustrated by SDS-PAGE (Figure 4A), we cannot dismiss the possibility of a lower content of specific proteins that typically influence digestibility in pulses, such as protease inhibitors [21,29]. Additional research is necessary to elucidate the intricate mechanisms involved in the digestion and protein hydrolysis of biscuit that integrates NC1 chickpea flour. In the gastrointestinal digestion, the use of a mixture of proteolytic enzymes results in hydrolysis products that are a heterogeneous mixture of small oligopeptides and free amino acids. The protein profile after the IVGD (Figure 4B) revealed an identical profile for all the samples. All proteins with an apparent molecular weight >30 kDa were digested, although certain proteins exhibited resistance during the digestion process. The polypeptide of relative molecular mass (Mr) ~25,000 evident in all digested samples in Figure 4B corresponds to protein PA2, which is resistant to the digestion process [21,30]. As anticipated, the digestion process resulted in a substantial increase in polypeptides of smaller amounts.

Protein quality involves not only the amino acid profile but also factors like bioavailability and digestibility, facilitating the absorption of amino acids. Therefore, the free amino acid profile at the conclusion of digestion is a crucial parameter for assessing a food protein’s ability to meet metabolic demands for amino acids. The free amino acids quantified from the digestion of the biscuits supported the greater DH of the NC1 samples, with this biscuit providing higher amounts of most amino acids. The gastrointestinal digestion of biscuits revealed arginine as the most abundant non-essential amino acid, while essential amino acids such as phenylalanine, leucine, and lysine were predominant. This amino acid composition in biscuit formulations could be viewed as an enhanced nutritional feature, given that the intake of branched-chain amino acids (specifically leucine, isoleucine, and valine) has been associated, among other factors, with a positive skeletal muscle mass index [31].

### 3.3. Antioxidant Properties and Phenolic Compounds: Effects of IVGD

The high antioxidant capacity in chickpea seeds have been associated with the presence of polyphenolic compounds, which are mainly stored under the seed coat. During the first hydration phase of seed germination, various components of the ROS-mediated signal pathways are activated and accumulated. The final stress resistance degree can be attributed to the permanence of these antioxidant mechanisms activated in seeds [32]. In the undigested samples, the Control biscuit consistently exhibited lower TPC and antioxidant activity values than the biscuits supplemented with chickpea flours (*p* < 0.05), with those formulated with non-commercial seeds (NC1 and NC2) being particularly noteworthy (*p* < 0.05) (Table 2).

Phenolic compounds contribute to the overall antioxidant activities of plant foods owing to their capability to remove free radicals, chelate metal catalysts, activate antioxidant enzymes, and inhibit oxidases [33], although other compounds or pigments could also contribute. The darkness of legumes is correlated with their phenolic content and antioxidant activity measured using the FRAP and ORAC methods [33,34]. Similarly, our results showed significant correlations among the darkness of the biscuits and TPC and antioxidant values (Table 3). In addition to the effect of the chickpea flour’s color, another reason may be the formation of Maillard reaction products during baking, i.e., brown products or melanoidins, which act as antioxidant compounds with scavenge-free radical capacity. In line with our results, the study conducted by Saeed et al. [35] further supports the idea that the incorporation of roasted chickpea flour into wheat biscuits results in elevated values of both FRAP and TPC. Notably, as the proportion of chickpea flour in the recipe increased, the improvements in these antioxidant parameters became more pronounced. The positive effect of the incorporation of chickpea flour in oat milk by-product-based biscuits on the antioxidant properties measured using the ABTS method has also been described [36]. These combined findings emphasize the opportunity to enhance the nutritional profile and antioxidant attributes of biscuits by incorporating chickpea flour.

Nevertheless, a more in-depth analysis is needed to evaluate the results following the digestion process. The IVGD procedure, in this context, resulted in a three- to four-fold increase in TPC values and antioxidant activity, as measured via the FRAP procedure, for all of the biscuits (Table 2). When the ABTS method was employed, the increase in the antioxidant action was even more pronounced. It is important to mention that the ABTS is a synthetic radical not existing in vivo; however, it provides information on the antioxidant compounds’ possible behavior in the organism. After digestion, biscuits supplemented with chickpea flour showed higher TPC and antioxidant activity compared to the Control biscuit (*p* < 0.05). The pattern exhibited was similar to that described for the undigested samples with the only deviation of the ABTS values observed in biscuits made with commercial chickpeas (C1 and C2), which did not significantly differ from the traditional shortbread biscuit. The statistically higher FRAP values observed in these samples compared to the control imply the formation of antioxidant compounds post digestion. The mechanism of action of these compounds appears to be mediated by metal-reducing activity rather than free radical scavenging. As far as we know, there is no scientific literature describing the effect of IVGD on the antioxidant activity and TPC of chickpea flour-enriched biscuits. Studies on pasta incorporating increasing proportions of chickpea flour along with chia reveal that while cooking has a negative impact on phenolic content and antioxidant capacity, the digestive process effectively counteracts these effects. This resulted in the preservation of increased levels of phenolic compounds and antioxidant capacity in the BFs, maintaining their potential health benefits [37].

**Figure 5:**
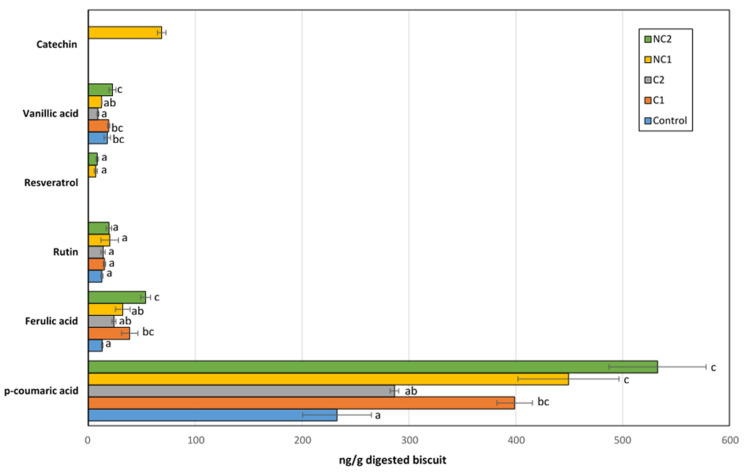
Major phenolic compounds detected in the bioaccessible fraction after IVGD of formulated biscuits. Data are expressed as mean values ± SD. Different letters within each compound indicate significant differences between biscuits (*p* < 0.05).

The identified free phenolic compounds in the BFs of biscuits included p-coumaric, ferulic, and vanillic acids, along with rutin, resveratrol, and catechin (Figure 5). The latter was only found in the BF of chickpeas made with the NC1 flour. Although not all the phenolics present in the seeds are bioaccesible, the profile found in this fraction was clearly dependent on the seed used in the biscuit composition. Only rutin, a glycoside obtained by combining the flavonol quercetin and the disaccharide rutinose, was found in the same amount in all samples, so we cannot rule out the possibility that this could originate from other ingredients in the recipe, rather than chickpea flours. Interestingly, rutin has been detected in corn seeds [38], which was part of the biscuit formulation.

Compared to the Control biscuits, all chickpea biscuits exhibited higher levels of p-coumaric acid (especially in NC1, NC2, and C1; *p* < 0.05), which was the predominant phenolic acid among all detected. Ferulic acid was the second most abundant, with concentrations 10 times lower than that of p-coumaric acid. Similar to the trend observed with p-coumaric acid, the BF of chickpea biscuits showed an enrichment in ferulic acid compared to the Control biscuit. Therefore, the inclusion of chickpea flours in the formulation provided additional amounts of these acids after gastrointestinal digestion, whose antioxidant, anti-inflammatory, antimutagenic, or anti-cancer activities are well-recognized [39,40]. Fares and Menga [41] described the presence of significant amounts of free ferulic and p-coumaric acids in the Sultano variety. On the other hand, both the thermal processing and the gastrointestinal process could also release bound phenolic acids, thus contributing to the free fraction of p-coumaric and ferulic acids detected after IVGD [42]. Resveratrol is a non-flavonoid polyphenol which has been largely related to beneficial health effects, including anticancer, antimicrobial, neuroprotective, antiaging, anti-inflammatory, cardioprotective, and blood-sugar lowering properties, as well as life-prolonging actions [43]. Although chickpeas are not considered a source of resveratrol in diets, low amounts of this compound were detected after the IVGD of biscuits containing non-commercial chickpea seeds (NC1 and NC2). Previous studies have revealed important levels of its condensed form in chickpea shoot powder, which could be partially released after digestion [44]. Finally, a remarkable catechin concentration was measured in the BF of the NC1 biscuit, with a level higher than the rest of the phenolics except for p-coumaric acid. This carries significant implications for its antioxidant activity, as this phenolic acid, previously described in other chickpea ecotypes, has exhibited therapeutic effects through its antioxidant, anti-inflammatory, or anti-apoptotic properties [45,46].

The relationships between variables defining the antioxidant profile of the formulated biscuits were assessed by calculating Pearson’s linear correlation coefficient (Table 3).

The Folin–Ciocalteu reactive used in the TPC determination is quite unspecific, and it is known that compounds other than phenolics, such as proteins and sugars with a reducing power that might be present in chemical extracts of biscuits and their BFs, could interact with it [47]. Besides this, TPC showed significant and positive correlations with ABTS and FRAP in undigested biscuits, and ABTS and FRAP were also correlated themselves. The correlation of TPC-FRAP persisted after the IVGD of biscuits, unlike TPC-ABTS, suggesting a mechanism involving metal-reducing activity rather than free radical scavenging among the antioxidant compounds released into the BFs. Similarly, p-coumaric and ferulic acids showed positive correlations with TPC, FRAP, and ABTS values in undigested biscuits, but this correlation was lost for ABTS after IVGD. Notably, both acids exhibited significant and positive correlations between each other. In summary, incorporating chickpea flour into the biscuit recipe improved the antioxidant activity and TPC in both undigested samples and their BFs. This enrichment led to higher levels of p-coumaric and ferulic acids, regardless of the type of chickpea flour used. Furthermore, non-commercial chickpea flours tested led to an enrichment in resveratrol and/or catechin.

### 3.4. ROS Generation in Caco-2 Cells

**Figure 6:**
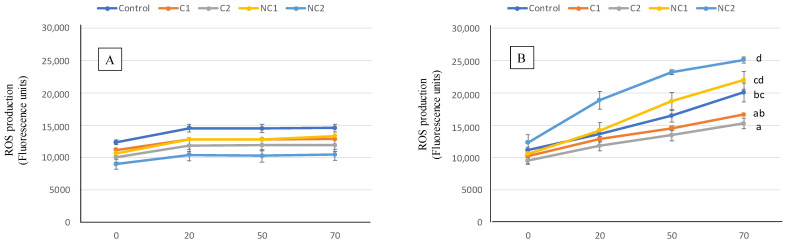
ROS generation expressed as fluorescence units for 70 min in Caco-2 cells pre-incubated with BF of the formulated biscuits compared with control cells incubated with culture medium. (**A**) Basal conditions. (**B**), oxidative stress induced with t-BOOH 20 mM. Data are expressed as mean values ± SD. Different letters at each time indicate significant differences between biscuits (*p* < 0.05).

The antioxidant activity of the digested samples was also assessed at the cell level by measuring the effect in ROS production of Caco-2 cells (Figure 6). At basal or physiological conditions, there is always a slight production of ROS by cells, a consequence of their normal metabolism being able to properly trigger defense metabolic pathways [48]. In such situations (part A), preincubating the cells for 2 h with the BF of the biscuits induced modest ROS generation, with no differences found among the different biscuits. When an oxidative injury was induced, the ROS production was strongly stimulated, resulting in stressed cells (part B). Then, differences in the ROS final level were observed after exposure to digested biscuits, thus indicating diverse capacities to prevent the free radical overproduction. In this case, the prevention effect was more pronounced with samples containing commercial chickpea flour, whereas the conventional biscuit (Control) showed intermedium ROS induced levels between C and NC chickpea samples. 

Usually, the antioxidant properties of chickpeas or food preparations containing chickpeas have been evaluated using in vitro methods (ABTS, DPPH, or FRAP), using chemical extracts obtained from organic solvents [35,49] or fractions from the digestive process [37], but information about the effect on antioxidant cell markers is very scarce. Cell cultures offer the possibility to study interactions between nutrients and cellular structures or metabolic pathways, thus providing results of a high biological significance. Our results in Caco-2 cells were surprising, since cell antioxidant activity did not correlate with TPC or in vitro antioxidant markers of ABTS and FRAP. However, although phenolic compounds are considered as the principal antioxidants in cereals and legumes, other phytochemicals such as vitamin E, phytic acid, carotenoids, glucans, and lignans also show strong antioxidant ability [36]. Glucans are mainly located in starch, and it has been shown that incubating Caco-2 cells with glucans decreased ROS levels [50]. On the other hand, some phenolic compounds, such as vainillic acid, show antioxidant capacity when chemically measured but are inefficient in protecting cells of induced oxidative damage [51], suggesting that phenol’s structure is crucial to exert a protecting effect at the biological level. In addition, small peptides resulting from the protein hydrolysis during digestion may act as active antioxidant at the cell level [52], as it has been observed from measuring the ROS production in Caco-2 cells incubated with lupin protein hydrolysates [53].

According to our results and as previously reported by others, although in vitro antioxidant assays are based on well-known chemical reactions, these probably do not reflect the cellular physiological environment, and, therefore, the in vitro antioxidant capacity of a compound or food matrix does not always correspond to its biological antioxidant protective effect [51].

## 4. Conclusions

The incorporation of chickpea in the formulation of biscuits resulted in improved traits for consumer acceptance, like appearance and texture. But, more importantly, it improved its nutritional value through higher amounts of proteins. The in vitro digestion process revealed good digestibility values. In this sense, it is important to highlight that the type of seed used will have an impact on the nutritional characteristic of the final product. In our case, seeds NC1 had an apparent higher protein hydrolysis after digestion and, consequently, a higher amino acid released. Chickpea flour also improved the antioxidant action after IVGD; again, the type of seed influenced the profile of polyphenols found in the bioaccessible fraction. Surprisingly, the increased antioxidant ability found after IVGD was not translated into a higher protection in cells, highlighting the importance of deeper studies in this sense before declaring any health claims. Further studies are being carried out in our laboratory, exploring the colonic fermentability of biscuits and the influence of the flour types used.

## Figures and Tables

**Table 1:** Protein content in formulated biscuits, degree of hydrolysis (DH) after IVGD, and amino acid composition before and after IVGD.

Biscuits								
Amino Acids	C1		C2		NC1		NC2	
Protein (%)	7.73 ± 0.07 ^a^		8.23 ± 0.15 ^a^		8.99 ± 0.37 ^b^		9.69 ± 0.03 ^c^	
DH (%)	15.03 ± 2.12 ^a^		15.38 ± 1.88 ^a^		19.78 ± 1.60 ^b^		15.48 ± 1.10 ^a^	
Essential (mg/g biscuit)	Before	After	Before	After	Before	After	Before	After
His	1.55 ± 0.09 *	0.23 ± 0.01 ^a^	1.74 ± 0.03 *^†^	0.31 ± 0.04 ^ab^	1.78 ± 0.03 *^†^	0.34 ± 0.05 ^c^	1.92 ± 0.11 ^†^	0.26 ± 0.03 ^ab^
Ile	2.59 ± 0.10	0.46 ± 0.07 ^a^	2.92 ± 0.02	0.63 ± 0.00 ^a^	2.94 ± 0.07	0.62 ± 0.09 ^a^	2.80 ± 0.20	0.41 ± 0.02 ^a^
Leu	4.23 ± 0.17	1.69 ± 0.10 ^ab^	4.76 ± 0.08	1.98 ± 0.12 ^bc^	4.74 ± 0.11	2.11 ± 0.04 ^c^	4.69 ± 0.32	1.55 ± 0.10 ^a^
Lys	4.09 ± 0.22	1.23 ± 0.07 ^a^	4.53 ± 0.08	1.59 ± 0.02 ^c^	4.59 ± 0.09	1.50 ± 0.09 ^bc^	4.62 ± 0.23	1.31 ± 0.07 ^ab^
Met ^1^	1.28 ± 0.07	0.41 ± 0.06 ^a^	1.44 ± 0.03	0.49 ± 0.08 ^a^	1.35 ± 0.03	0.51 ± 0.11 ^a^	1.33 ± 0.07	0.36 ± 0.04 ^a^
Phe	3.48 ± 0.14	2.57 ± 0.15 ^a^	3.94 ± 0.09	2.97 ± 0.09 ^bc^	4.01 ± 0.09	3.29 ± 0.12 ^c^	3.85 ± 0.31	2.69 ± 0.11 ^ab^
Thr	2.13 ± 0.10	0.14 ± 0.01 ^a^	2.37 ± 0.04	0.23 ± 0.01 ^a^	2.27 ± 0.06	0.25 ± 0.13 ^a^	2.34 ± 0.09	0.16 ± 0.04 ^a^
Val	2.71 ± 0.11	0.51 ± 0.04 ^ab^	3.01 ± 0.01	0.66 ± 0.10 ^ab^	3.06 ± 0.06	0.77 ± 0.08 ^b^	2.93 ± 0.19	0.45 ± 0.10 ^a^
Non-essential (mg/g biscuit)								
Ala	2.51 ± 0.12	0.47 ± 0.04 ^ab^	2.82 ± 0.04	0.59 ± 0.06 ^b^	2.81 ± 0.07	0.56 ± 0.08 ^b^	2.84 ± 0.15	0.35 ± 0.07 ^a^
Arg	5.82 ± 0.34 *	3.49 ± 0.20 ^a^	6.55 ± 0.08 *	4.15 ± 0.08 ^b^	8.07 ± 0.20 ^†^	5.07 ± 0.03 ^c^	9.12 ± 0.34 ^†^	5.36 ± 0.11 ^c^
Asp ^2^	6.89 ± 0.31	0.04 ± 0.00 ^b^	7.79 ± 0.23	0.13 ± 0.02 ^b^	7.82 ± 0.13	0.14 ± 0.00 ^bc^	7.81 ± 0.40	0.19 ± 0.01 ^c^
Cys ^3^	0.82 ± 0.04	0.41 ± 0.03 ^b^	0.96 ± 0.01	0.54 ± 0.02 ^c^	0.82 ± 0.02	0.37 ± 0.01 ^b^	0.87 ± 0.05	0.28 ± 0.03 ^a^
Glu ^4^	9.64 ± 0.67	0.71 ± 0.07 ^a^	10.89 ± 0.13	0.85 ± 0.07 ^bc^	11.04 ± 0.30	0.98 ± 0.08 ^c^	11.51 ± 0.60	0.74 ± 0.10 ^bc^
Gly	2.39 ± 0.11	0.09 ± 0.01 ^ab^	2.68 ± 0.05	0.15 ± 0.01 ^a^	2.66 ± 0.05	0.15 ± 0.05 ^b^	2.67 ± 0.12	0.02 ± 0.01 ^a^
Pro	2.44 ± 0.11	-	2.73 ± 0.05	-	2.81 ± 0.08	-	2.79 ± 0.14	-
Ser	2.77 ± 0.18	0.79 ± 0.05 ^ab^	3.09 ± 0.04	1.04 ± 0.02 ^bc^	3.12 ± 0.12	1.15 ± 0.11 ^c^	3.35 ± 0.12	0.61 ± 0.09 ^a^
Tyr	1.96 ± 0.07	1.68 ± 0.13 ^a^	2.19 ± 0.05	2.05 ± 0.13 ^ab^	2.15 ± 0.05	2.14 ± 0.22 ^b^	2.09 ± 0.11	1.61 ± 0.13 ^a^

^1^ Methionine was measured as methyl sulfone. ^2^ Asp included aspartic acid and asparagine. ^3^ Cysteine was measured as cysteic acid. ^4^ Glu included glutamic acid and glutamine. Data are mean ± SD (*n* = 3). For protein content and DH: different letters in the same row indicate significant differences between biscuits. For amino acids: (1) before digestion: different symbols (* and ^†^) in the same row indicate significant differences between biscuits; (2) after digestion: different letters in the same row indicate significant differences between biscuits. One-Way ANOVA followed by Tukey HDS test (*p* < 0.05).

**Table 2:** Total phenolic compounds (TPCs) and antioxidant activity of formulated biscuits before and after IVGD.

Biscuit	TPC (µmol GAE/g Biscuit)		ABTS (µmol TE/g Biscuit)		FRAP (µmol TE/g Biscuit)	
	Before	After	Before	After	Before	After
Control	9.91 ± 0.18 ^a^	27.90 ± 1.00 ^a^	2.39 ± 0.02 ^a^	138.23 ± 3.02 ^b^	1.76 ± 0.01 ^a^	5.66 ± 0.12 ^a^
C1	12.69 ± 0.09 ^b^	40.29 ± 0.61 ^b^	4.94 ± 0.16 ^c^	122.82 ± 7.00 ^ab^	3.94 ± 0.12 ^b^	12.90 ± 0.67 ^b^
C2	12.71 ± 0.14 ^b^	44.25 ± 0.52 ^b^	3.76 ± 0.20 d ^b^	108.36 ± 4.23 ^a^	3.66 ± 0.03 ^b^	18.27 ± 0.04 ^c^
NC1	14.91 ± 0.04 ^c^	53.76 ± 1.46 ^d^	12.95 ± 0.02 ^d^	164.25 ± 5.71 ^c^	12.06 ±0.12 ^c^	24.36 ±0.42 ^d^
NC2	14.84 ± 0.17 ^c^	49.17 ± 2.05 ^c^	14.73 ± 0.16 ^e^	141.53 ± 10.18 ^bc^	12.76 ± 0.30 ^d^	37.11 ± 2.79 ^e^

Data are mean ± SD (*n* =3). Different letters in the same column indicate significant differences between biscuits (*p* < 0.05). One-Way ANOVA followed by Tukey HSD test.

**Table 3:** Statistically significant correlations found between different parameters.

Parameters ^1^	R-Value ^2^	*p*-Value ^2^
L*—TPC_B_	−0.9687	0.0000
L*—ABTS_B_	−0.8191	0.0038
L*—FRAP_B_	−08269	0.0032
TPC_B_—ABTS_B_	0.8906	0.0005
TPC_B_—FRAP_B_	0.9068	0.0003
ABTS_B_—FRAP_B_	0.9961	0.0000
TPC_B—_TPC_A_	0.9745	0.0000
ABTS_B—_ABTS_A_	0.6579	0.0386
FRAP_B—_FRAP_A_	0.9021	0.0004
TPC_B_—p-coumaric acid_A_	0.9008	0.0143
ABTS_B_—p-coumaric acid_A_	0.9577	0.0026
FRAP_B_—p-coumaric acid_A_	0.9429	0.0048
TPC_B_—ferulic acid_A_	0.9045	0.0312
ABTS_B_—ferulic acid_A_	0.8973	0.0153
FRAP_B_—ferulic acid_A_	0.8800	0.0207
TPC_A_—FRAP_A_	0.8223	0.0035
TPC_A_—p-coumaric acid_A_	0.7894	0.0066
FRAP_A_—p-coumaric acid_A_	0.8697	0.0243
TPC_A_—ferulic acid_A_	0.6403	0.0461
FRAP_A_—ferulic acid_A_	0.8923	0.0168
Ferulic acid_A_—p-coumaric acid_A_	0.8783	0.0018

^1^ Subscript meaning: B, before IVGD; A, after IVGD. ^2^ Relationships were evaluated using the Pearson’s linear correlation coefficient with a significance level fixed at *p* < 0.05.

## Data Availability

The data presented in this study are available on request from the corresponding author.
